# Is it a rights violation or lack of knowledge about options? An examination of HIV counselors views on whether women infected with HIV should procreate

**DOI:** 10.11604/pamj.2013.14.79.1468

**Published:** 2013-02-27

**Authors:** Amos Kankponang Laar

**Affiliations:** 1 Department of Population Family and Reproductive Health, School of Public Health, College of Health Sciences, University of Ghana, Legon, Accra, Ghana

**Keywords:** Reproductive options, reproductive rights, HIV, service providers, Ghana

## To the editors of the Pan African Medical Journal

Prior to the widespread availability of antiretroviral therapy (ART), the desire to conceive among HIV-positive women was discouraged due to a high perinatal transmission risk [1]. The recent advancement in HIV therapy has transformed the conceptualization of the infection. In particular, highly effective prevention strategies have led to a near elimination of the pediatric HIV epidemic in the United States and Europe [2, 3]. “Virtual elimination”of pediatric HIV infection is a now touted as a near-term target [4], and the proportion of HIV-positive individuals who desire to exercise their fundamental right to reproduction is increasing.

With this reality, experts currently advise that health professionals to approach the HIV-positive client as a person with rights just as any other individual [5, 6]. As to whether the Ghanaian healthcare provider is abreast of the fact that HIV-positive persons have reproductive rightsmotivated the investigation from which this commentary metamorphosed. I examine in this short communication how knowledgeablea group of Ghanaian healthcare workers are on the subject of HIV and reproductive rights.

**Figure 1:**
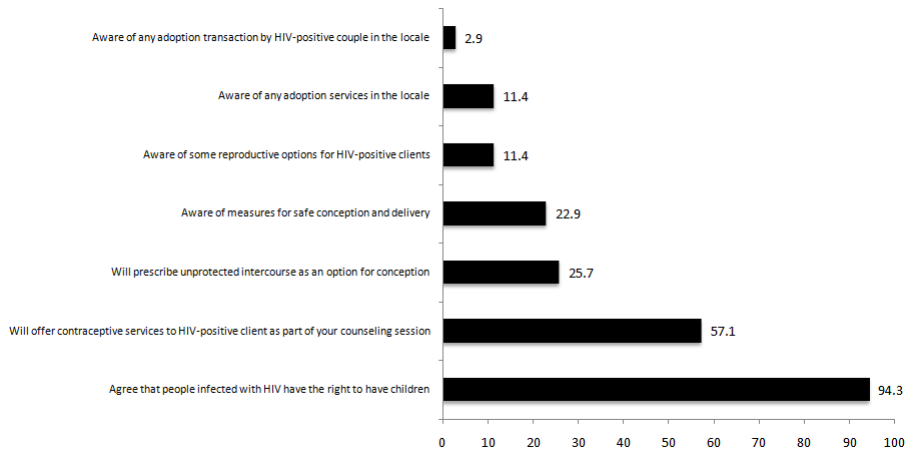
Perspectives of service providers on reproductive rights and options available to women infected with HIV (horizontal bars represent proportions; n = 35)

A survey of nurse counselors (32) and medical officers (3) providing counseling and testing services to HIV-positive clients in two Ghanaian districts was completed. Figure 1 depicts the results obtained. The study uncovers two main perspectives. On the one hand, there was an overwhelmingly high level of approbation by the providers on HIV-positive women's right to reproduction (94.3%), on the other providers demonstrated a disappointingly high level of ignorance regarding the various reproductive options available to women infected with HIV in Ghana. It is worthy of note that Articles 1 and 16 of the Universal Declarations of Human Rights recognize the reproductive rights of all humans. *(“All human beings are born free and equal in dignity and rights...”)...(“men and women of full age, without any limitation, have the right to marry and to found a family...”)* [7].Women infected with the AIDS virus clearly fall within this scope.Only about 10% of theproviders were aware of some reproductive options for HIV-positive women. A quarter would advise HIV-positive women to have unprotected intercourse as an option to conceive. Some of the providers openly expressed their inability to give qualified and relevant advice to HIV-positive women on the options tabled for discussion. There are no specific policies with regard to reproductive rights and options for HIV persons accessing services in Ghanaian hospitals. Other constraints mentioned were lack of resources and knowledge upgrade refresher trainings.In a related study in Tanzania, Leshabari et al. revealed a high level of stress, frustration, and acknowledgment of incompetence by the nurse-counselors [8].

In 2004, more than 25 national and international organizations presented a statement to the secretariat of the United Nations Commission on the Status of Women that highlighted relatively neglected areas in the reproductive health of women affected by HIV and AIDS [9]. Ipas in collaboration with the International Community of Women Living with HIV, the Center for Health and Gender Equity, and the Pacific Institute for Women's Health used that statement and a literature review [9] to develop a practical tool to help interested organizations address those neglected areas of reproductive health. Delineated in this tool are various reproductive options. It is very likely that most Ghanaian health workers are unaware of this resource.

In fact, anecdotes have long hinted on the need to strengthen existing service provision guidelines and to equip service providers with the requisite competencies to interact with HIV-positive clients professionally. In the case of HIV, it is not only the medical facts that are relevant to their health, but also many of the psychosocial issues relevant to their interest in having children [10]. Health workers themselves have sent out several calls for knowledge updates through refresher training. Owing to the frequency of such calls, one does not have to peruse the yearly reports churned out by the Ghana Health Servicefor this to be recognized. A fundamental premise for successful counseling is that the counselor has both confidence in his own professional knowledge, and the relevant application of this knowledge for the individual client being counseled. The Ghanaian counselor has acknowledged in this study, that he does not have the professional knowledge required to be successful.

These findings suggest that HIV-positive clients do not receive comprehensive information about their reproductive options. This may be (legitimately) blamed on ignorance. An alternative counseling approach that respects clients? rights to informed and considered decision-making concerning childbearing should be encouraged. Unfortunately, this is not possible without the revision and introduction of reproductive health rights arguments into the current service provision guidelines.Fortunately, as it stands now, it seems to me, it is not a rights denial, but a lack of options.

Although Ghanaian health care workers acknowledge the reproductive rights of HIV positive women, they are lacking the knowledge to adequately council women on their reproductive options.This calls for the recognition of the current realities regarding HIV and reproduction, and an urgent incorporation of reproductive health issues into existing local HIV policies and guidelines. Appropriate knowledge upgrade through refresher training could improve service provision to HIV-positive clients in Ghana.
